# Perioperative Hemostasis Management During Open Mitral Valve Replacement in a Patient With Hypoprothrombinemia Secondary to Lupus Anticoagulant

**DOI:** 10.7759/cureus.81472

**Published:** 2025-03-30

**Authors:** Obieze Nwanna-Nzewunwa, Tayyaba M Malik, Melissa Bellomy, Yu-Min Shen, Siavosh Saatee, Christopher Heid

**Affiliations:** 1 Cardiovascular and Thoracic Surgery, University of Texas Southwestern Medical Center, Dallas, USA; 2 Anesthesia and Critical Care, University of Texas Southwestern Medical Center, Dallas, USA; 3 Hematology, University of Texas Southwestern Medical Center, Dallas, USA

**Keywords:** antiphosphoplipid syndrome, cardiac surgery, hypoprothrombinemia, lupus anticoagulant, mitral valve replacement, perioperative management

## Abstract

Hypoprothrombinemia is a rare coagulopathy that complicates surgical interventions and is an uncommon manifestation of lupus anticoagulant. Perioperative coagulopathy is a major challenge in cardiac surgery. To our knowledge, the management of cardiac surgery in patients with hypoprothrombinemia has not been described in prior literature. This case report outlines the challenging surgical intervention of mitral valve replacement in a 47-year-old female patient with rheumatic valve disease (RHD) and a rare prothrombin deficiency secondary to lupus anticoagulant. This report details the multidisciplinary perioperative hemostasis management and outcomes.

## Introduction

Hypoprothrombinemia is one of the rarest coagulopathies and a complication of lupus anticoagulant that complicates surgical interventions [1,2]. Lupus anticoagulant (LA) is an autoimmune immunoglobulin that paradoxically increases thrombosis risk while also predisposing some patients to bleeding due to acquired prothrombin deficiency. Other causes include congenital prothrombin deficiency (autosomal recessive inheritance), vitamin K deficiency (due to malnutrition, malabsorption syndromes, and vitamin K antagonists like warfarin), liver disease (such as cirrhosis or hepatitis that impairs prothrombin production), antibiotics (e.g., cefazolin) that alter gut flora responsible for vitamin K synthesis, and chemotherapy agents [3].

A review of existing literature reveals that while prothrombin deficiency associated with lupus anticoagulant has been described in non-surgical settings, there is a lack of published reports detailing its perioperative management, particularly in the setting of cardiac surgery. Given the high risk of both thrombosis and catastrophic bleeding, the surgical approach to patients with lupus anticoagulant and hypoprothrombinemia is complex and poorly characterized. To the author's knowledge, this is the first report of cardiac surgery in a patient with hypoprothrombinemia due to lupus anticoagulant.

## Case presentation

A 47-year-old female presented with refractory heart failure due to rheumatic valve disease (RHD) with mixed mitral stenosis and regurgitation and atrial fibrillation on a background of prothrombin deficiency due to lupus anticoagulant, hypertension, seizure disorder, and chronic kidney disease stage 3 (estimated glomerular filtration rate (eGFR) 23 mL/min/1.73) and weighed 97 kg. She had several prior hospitalizations due to severe spontaneous bleeding episodes resulting in hemoperitoneum and hemopericardium requiring red blood cell transfusions. Although deemed high risk and prohibitive for mitral valve surgery by a prior surgeon due to her coagulopathy, our multidisciplinary evaluation of the patient informed a decision for open mitral valve replacement. 

Preoperative management

**Figure 1:**
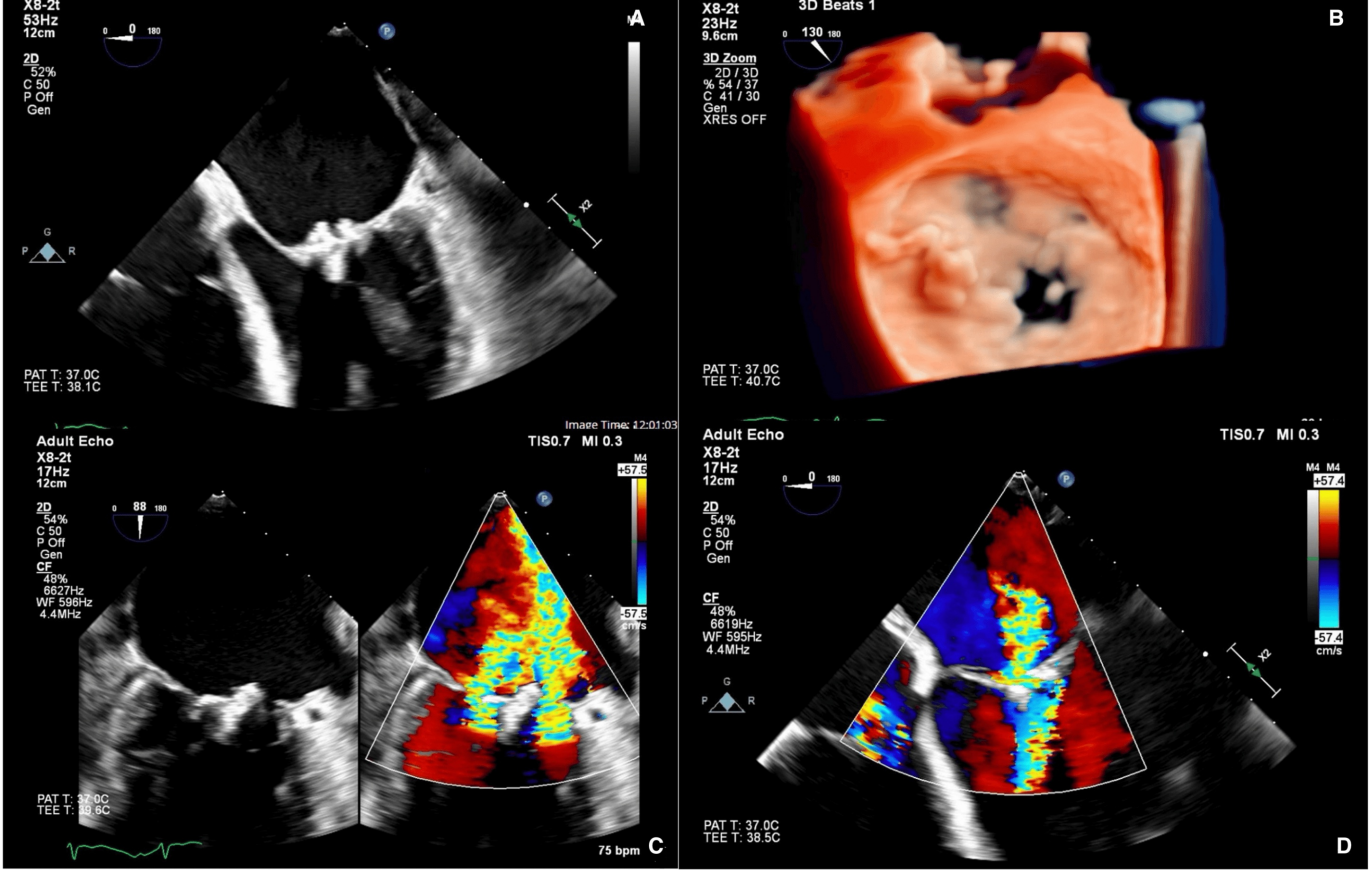
Preoperative transesophageal echocardiogram showing mitral valve vegetation and regurgitation. A: 2-dimensional view of mitral valve vegetation; B: 3-dimensional view of mitral valve vegetation; C, D: color Doppler view showing mitral valve regurgitation

Preoperative planning was done in collaboration with anesthesiology, hematology, cardiology, a cardiac intensivist, and cardiac surgery. Preoperative transesophageal echocardiogram (Figure 1) showed diffusely thickened and dysmorphic mitral leaflets, severe mitral regurgitation, moderate-to-severe mitral stenosis, and a left ventricular ejection fraction of 50% without other significant valvopathies. Staphylococcus epidermidis bacteremia prompted antibiotic treatment for possible endocarditis.

**Figure 2:**
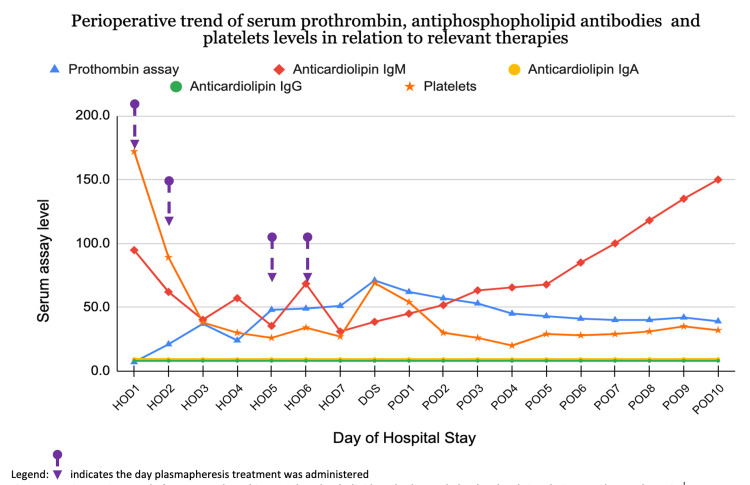
Perioperative trend of serum prothrombin, antiphospholipid antibodies, and platelet levels in relation to relevant therapies.

The preoperative hemoglobin ranged from 8.1 - 8.5g/dL, while the activated partial thromboplastin time (aPTT) ranged from 35 - 53.4 seconds. The outpatient prothrombin baseline level was 6% (reference range = 80-129%). Outpatient rituximab 850 mg weekly for four weeks aimed at achieving a prothrombin level of >30% failed to appropriately raise her prothrombin level and platelet count (normal range 150,000 - 450,000 μL). The platelet count went from 120 x 10^9^ to a peak of 172 x 10^9^/L two weeks after completing rituximab. After four plasmapheresis sessions, her prothrombin levels improved over 11-fold from 6% on the day of admission to 71% on the day of surgery (Figure 2); however, her platelet levels dropped from 172 x 10^9^ to 69 x 10^9^/L. The latter was attributed to autoantibodies (i.e., immune thrombocytopenia purpura) and treated with 45 g of intravenous immunoglobulins (IVIG) on the day of surgery, ahead of the procedure. Preoperatively, 1,600 units of four-factor prothrombin concentrate (Kcentra®) were administered to optimize prothrombin levels.

Operative details

**Figure 3:**
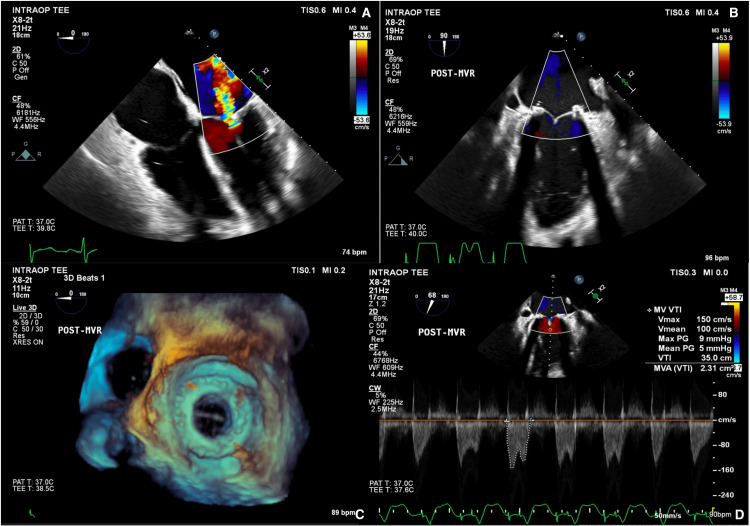
Intraoperative transesophageal echocardiography showing mitral valve anatomy before and after replacement and hemodynamics. A, B: intraoperative view of mitral valve before (A) and after (B) mitral valve replacement (MVR). C, D: 3D echocardiogram of the new 31 mm Mitris (Edwards Lifesciences Corporation, CA, USA) valve in the mitral position (C) and 2D echocardiogram and new valve with measurements.

Standard preparation, induction of anesthesia, intubation, and vascular access were achieved. Intraoperative transesophageal echocardiography (TEE) redemonstrated preoperative findings. A mitral valve replacement with a 31mm Edwards Mitris (Edwards Lifesciences Corporation, CA, USA) valve was performed under cardiopulmonary bypass via median sternotomy. Heparinization (30,000 units) achieved an activated clotting time of 750 seconds from a baseline of 112 seconds, which was ultimately reversed with 300 mg of protamine. TEE showed peak and mean transmitral gradients of 10 mmHg and 3 mmHg, respectively, and no paravalvular leaks (Figure 3). Left ventricular ejection fraction (LVEF) was 40%.

Intraoperatively, she received three units of packed red blood cells (PRBC). Desmopressin (35.6 mg) was given with a six-pack of platelets for a platelet count of 26 x 10^9^/L and decreased clot firmness on rotational thromboelastometry (ROTEM), two five-packs of cryoprecipitate for a fibrinogen level of 182 mg/dL and abnormal fibrin-based thromboelastometry (FIBTEM), and 1604 units of four-factor prothrombin complex concentrate (Kcentra®) and aminocaproic acid (5 mg). Hemostasis was adequate. 

Postoperative management

The postoperative course was unremarkable. She received a total of two PRBCs and three platelets postoperatively and was dialyzed once. Mycophenolate mofetil (1000 mg BID) was initiated to eliminate the antiprothrombin antibodies, and romiplostim (Nplate®) (475 mg every seven days) was to decrease the bleeding risk. She was not discharged on anticoagulant medication or antiplatelets. Suppressive antibiotic therapy (IV daptomycin 750 mg every 48 hrs) was given for six weeks from surgery. She was not discharged on anticoagulation and antiplatelet therapy.

Outcome

Aside from a brief readmission for volume overload on postoperative day 30, the patient experienced an uneventful postoperative course, demonstrating a significant improvement in functional status and the New York Heart Association (NYHA) score. Early follow-up revealed satisfactory functioning of the replaced mitral valve, with no major complications attributed to prothrombin deficiency or lupus anticoagulant.

## Discussion

This study demonstrates that cardiac surgery, specifically mitral valve surgery, is feasible and safe in a patient with hypoprothrombinemia with multidisciplinary care, planning, meticulous intraoperative technique, hemostasis, and judicious use of blood products and hemostatic agents.

Lupus anticoagulant (LA) is an autoantibody that targets phospholipid-binding proteins, like prothrombin, leading to an increased risk of thrombosis rather than bleeding [3]. Paradoxically, some patients develop lupus anticoagulant hypoprothrombinemia syndrome (LAHPS), which causes bleeding [4]. LAHPS is a multifaceted coagulopathy involving both functional inhibition and antibody-mediated clearance pathways. Via the functional inhibition pathway, LA directly affects the coagulation cascade by hindering thrombin formation, thus reducing clot stability [5,6]. Through the antibody-mediated clearance pathway, antibodies bind to prothrombin without inactivating it but enhance the clearance of the antibody-prothrombin complex, thus reducing the available prothrombin pool, thus compounding the effects of the functional inhibition [5,6,7]. Both pathways can result in a bleeding tendency, despite the general thrombotic risk associated with lupus anticoagulant [8]. LAHPS can also occur in patients who test negative for prothrombin antibodies [7]. Hence, a careful and balanced perioperative management approach, including the use of immunosuppressive therapies, plasma exchange, and meticulous intraoperative and postoperative coagulation monitoring, is required to mitigate the risks associated with both thrombosis and bleeding in surgical patients.

Options for monitoring coagulation in LAPHS include thromboelastography (TEG), rotational thromboelastometry (ROTEM), and Quantra Hemostasis system (HemoSonics, LLC, Durham, North Carolina, USA), which are excellent tools for assessing functional hemostasis but do not precisely identify specific factor deficiencies. A comprehensive assessment of the clotting process should include anti-Xa levels for patients on heparin or low molecular weight heparin, and prothrombin time (PT) and international normalized ratio (INR) for overall coagulation status, although interpretation should be cautious due to the interference of lupus anticoagulant. Activated partial thromboplastin time (aPTT) should be monitored with baseline comparisons, while fibrinogen levels, platelet count and function, D-dimer levels, and prothrombin time mixing studies provide further insights into the coagulation status. Direct measurement of factor II (prothrombin) activity is crucial for assessing the severity of the deficiency and guiding replacement therapy. Perioperative management may require administering fresh frozen plasma (FFP), cryoprecipitate, or specific coagulation factor concentrates if significant bleeding occurs. In this case, direct thrombin replacement for her hypoprothrombinemia was primarily achieved through prothrombin complex concentrate, and fresh plasma and cryoprecipitate were used specifically to address the anticipated hypofibrinogenemia following cardiopulmonary bypass. 

Our goal was to reverse the hypoprothrombinemia and high antiprothrombin levels preoperatively. Rituximab had a limited effect on this patient and others in a retrospective case series [9]. Plasmapheresis was more effective in improving prothrombin levels and decreasing IgM anti-prothrombin levels (Figure 2) but had no effect on IgG anti-prothrombin. A future therapeutic option for decreasing antithrombin IgG is the neonatal fragment crystallizable receptor (FcRn). FcRn functions as a recycling mechanism, preventing degradation and extending the half-life of IgG [10]. Several FcRn inhibitors, which selectively target IgG recycling and thus accelerate IgG clearance, have shown promising results in recent clinical trials for managing immune thrombocytopenia (ITP) and myasthenia gravis [11]. Given the similarity in the pathogenesis pathway, this strategy could be considered as a potential management approach in future cases [10].

Adequate platelet count and function are required for hemostasis. Thrombocytopenia in this patient was attributed to ITP and treated with PLEX, romiplostim, and platelet transfusions, resulting in an increase of platelet count to 69 x 10^9^/L from the 20 x 10^9^/L - 40 x 10^9^/L range. Lupus anticoagulant, daptomycin, and romiplostim can cause ITP thrombocytopenia [12,13]. Platelet function tests such as platelet function analyzer (PFA-100, Siemens Healthineers, Delaware, USA), light transmission aggregometry (LTA), and VerifyNow (Instrumentation Laboratory India Pvt Ltd., Haryana, India) may help in assessing bleeding risk, guiding perioperative management, and monitoring the effectiveness of therapies like intravenous immunoglobulin (IVIG) and romiplostim. By providing real-time data on platelet performance, these tests enable timely and targeted interventions, balancing the risks of thrombosis and bleeding to ensure safer surgical outcomes.

## Conclusions

This case highlights the successful perioperative management of a rare coagulopathy-hypoprothrombinemia secondary to lupus anticoagulant during open mitral valve replacement. Given the high bleeding risk and limited precedent in the literature, our multidisciplinary approach demonstrates a structured strategy for optimizing coagulation parameters preoperatively, ensuring intraoperative hemostasis, and managing postoperative anticoagulation. This case underscores the importance of individualized, protocol-driven management in patients with complex coagulopathies, providing a potential framework for similar high-risk surgical cases in the future.
